# The relationship between body mass index and preeclampsia: A systematic review and meta-analysis

**DOI:** 10.18502/ijrm.v17i7.4857

**Published:** 2019-07-31

**Authors:** Morteza Motedayen, Mohammad Rafiei, Mostafa Rezaei Tavirani, Kourosh Sayehmiri, Majid Dousti

**Affiliations:** ^1^ Department of Cardiology, Faculty of Medicine, Zanjan University of Medical Sciences, Zanjan, Iran.; ^2^ Department of Biostatistics and Epidemiology, School of Medicine, Arak University of Medical Sciences, Arak, Iran.; ^3^ Proteomics Research Center, Shahid Beheshti University of Medical Sciences, Tehran, Iran.; ^4^ Psychosocial Injuries Research Center, Department of Biostatistics, School of Public Health, Ilam University of Medical Sciences, Ilam, Iran.

**Keywords:** Preeclampsia, Body mass index, Iran, Meta-analysis.

## Abstract

**Background:**

One of the causes of maternal and fetal mortality and morbidity is pregnancy-induced hypertension, the most common form of which is preeclampsia that causes many complications for mother and fetus.

**Objective:**

The aim of this systematic review and meta-analysis was to determine the relationship between body mass index (BMI) and preeclampsia in Iran.

**Materials and Methods:**

Using valid keywords in the SID database, PubMed, Scopus, data obtained from all the articles, which were reviewed in Iran between 2000 and 2016, were combined using the meta-analysis method (random-effects model) and analyzed using STATA version 11.1.

**Results:**

A total number of 5,946 samples were enrolled in 16 studies with the mean BMI values of 25.13, 27.42, and 26.33 kg /m${}^{2}$ in the healthy, mild, and severe preeclamptic groups, respectively.

**Conclusion:**

The results of this study revealed that there is a significant relationship between BMI and the risk of preeclampsia, so it can be said that BMI may be one of the ways to diagnose preeclampsia.

## 1. Introduction

Preeclampsia is a pregnancy-related disease that increases maternal and perinatal mortality and morbidity (1, 2). The possible causes of preeclampsia include abnormal vascular thromboembolic invasion, lack of maternal-fetal immune tolerance and maternal maladaptation with cardiovascular and inflammatory changes during pregnancy and genetics (3). Preeclampsia affects 5–8% of all pregnancies (4) and causes many complications for mother and fetus in such a way that 50,000 women worldwide die from preeclampsia and its complications annually (5). Researchers believe that preeclampsia is a multifactorial disease and propose several risk factors for it, including a history of preeclampsia, low and high maternal age, diabetes, chronic hypertension, null parity, birth intervals, history of abortion, high body mass index (BMI) value, twin pregnancy, fetal sex, migraine, and maternal RH (6-10). A major part of the complications and some of the risk factors proposed for this disorder can be identified and prevented. Although the termination of pregnancy is considered as a definitive treatment of preeclampsia, careful prenatal care and appropriate treatment can improve the condition, and the outcome can be satisfactory for the mother and fetus in many cases (11). Some studies have referred to obesity as a risk factor for preeclampsia and showed that the relationship between maternal weight and preeclampsia is a progressive risk and varies from 4.3% in women with a BMI < 19.8 Kg/m${}^{2}$, up to 13.3% for women with a BMI ≥ 35 kg/m${}^{2}$ (12).

Since the objectives of meta-analysis include regular and systematic review of the evidence, the quantitative summing up of the results of each study, combining the results of various studies and providing a general interpretation of the results (13), the aim of the present study was to investigate the relationship between the maternal BMI and preeclampsia using the by meta-analysis technique, performing more studies in this area, and reducing neonatal morbidity and mortality.

## 2. Materials and Methods

The present study was carried out using the meta-analysis technique and random-effects models. It was conducted in several steps to accurately determine the problem under study and collecting, analyzing, and interpreting the findings and using preferred reporting items for systematic reviews and meta-analyses (PRISMA). The study began after using the aforementioned protocol as a research criterion as well as identifying the members of the meta-analysis group (including the group's supervisor and the final reviewer, the group's advisor, reviewer, and researcher; data extractor and collector; and article explorer). To review the studies, SID, Scopus, and PubMed databases were first systematically searched using valid Persian keywords (preeclampsia, BMI, pregnancy) and valid English keywords (preeclampsia, BMI, and pregnancy) in order to identify and evaluate the studies on the prevalence of preeclampsia in pregnant women in Iran. The studies were later classified as case-controls.

### Selection of studies and data extraction

All articles related to preeclampsia were collected and a list of articles' abstract was later prepared after the completion of the search. Then all articles, in which the BMI value was specified, were included in the first list.

Then, quality assessment was done by the Newcastle-Ottawa Scale. The adjusted Ottawa checklist was used to evaluate the quality of the studies. In this case, three subsets of the groups (four questions), group comparability (one question), and exposure or outcome (two questions) are examined (14), this tool is usually used for Validity assessment and is a reliable instrument with a long history of reliability (15).

**Figure 1:**
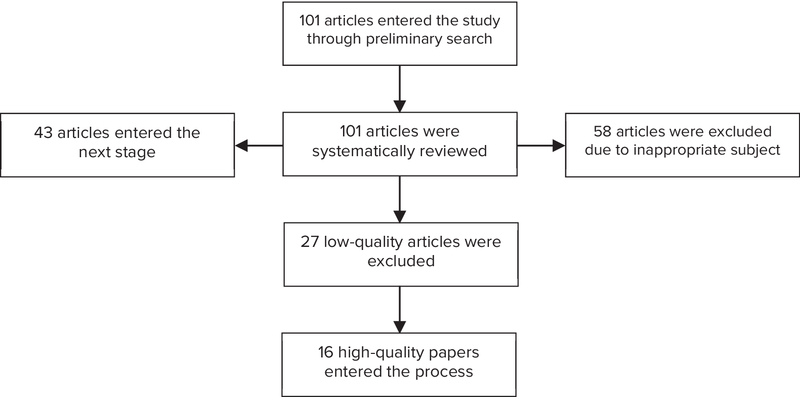
Flowchart of the stages of the systematic review and meta-analysis.

Studies with a score of 3 or more were considered qualitative and entered into the analysis. Other studies that addressed preeclampsia, causes of preeclampsia, ways to cope with preeclampsia, and risk factors for preeclampsia in pregnant women were excluded from the list. A necessary information checklist containing the researcher's name, article's title, year of conducting the research, research setting, code of the research setting, age group, sample size, BMI, etc., were prepared for all studies that underwent the initial evaluation in order to undergo the final evaluation. Finally, the final checklist was reviewed and the relevant articles were entered into the meta-analysis. According to these steps, 101 articles were found using the keywords in the first search. After the analysis phase, 16 appropriate articles entered the meta-analysis phase (Figure 1, Table I).

**Table 1:** Specifications of the studies performed


**Researcher**	**Year of study implementation**	**Sample size**	**Type of study**	**Average age group of participants**	**BMI in healthy group **	**95% CI (BMI in the healthy group)**	**BMI in mild PE group**	**95% CI (BMI in the mildly affected group)**	**BMI in the severe affected group**	**95% CI (BMI in the severe affected group)**
16	2007	200	Case-control	26.88	24	23.17–24.73	26	25.72–26.28	26	24.86–27.14
17	2012	498	Case-control	29.01	25.91	25.27–26.55	28.99	27.63–30.35	26.29	25.31–27.27
18	2007	400	Case-control	23.19	26.24	25.97–26.51	–	–	29.93	28.21–31.65
19	2012	112	Case-control	24.94	23.60	22.68–24.52	–	–	22.39	20.43–24.35
20	2010	675	Case-control	32.85	21.05	–	–	–	21.15	–
21	2006	636	Case-control	27	–	–	–	23	–
22	2010	610	Case-control	22.85	–	–	–	20	–
23	2013	90	Case-control	27.3	25.30	24.57–26.03	–	–	25.90	25.02–26.78
24	2009	187	Case-control	25.81	23.25	23.01–23.49	–	–	23.47	23.19–23.75
25	2008	64	Case-control	26.37	29.14	28.96–29.32	–	–	30.35	30.14–30.56
26	2010	60	Case-control	31.67	21.95	21.77–22.13	–	–	23.65	23.38–23.92
27	2001	674	Case-control	24.4	24.20	23.61–24.79	–	–	24	23.61–24.39
28	2010	60	Case-control	31.51	26.74	26.59–26.89	–	–	27.61	27.44–27.78
29	2006	80	Case-control	23.7	12.41	–	–	–	12.61	–
30	2010	1200	Case-control	28.11	25.45	–	–	–	28.56	–
BMI: Body mass index										

### Inclusion and exclusion criteria

Inclusion criteria included access to the full text of the article, English or Persian, articles aimed at examining the relationship between the components studied. Exclusion Criteria included unrelated articles, the inaccessibility of the full text of the article, and the incomplete abstract of the article. All analyzes were performed in this study using STATA according to the critical evaluation criteria and the eligible articles were included in the study.

### Statistical analysis

Considering that the main index studied in this review study was BMI, its variance was calculated by the binomial distribution, and the odd ratio with a 95% confidence interval (CI) was calculated. The weighted average was used to combine the rates obtained from different studies. Each study was weighted inversely in proportion to its variance. The heterogeneity was evaluated using Q test and I2 at the given significance level (α < 10%). Data analysis was performed using the meta-analysis technique (random-effects model) in cases of any heterogeneity. Data analysis was carried out using R and STATA Version 11.2.

## 3. Results

**Figure 2:**
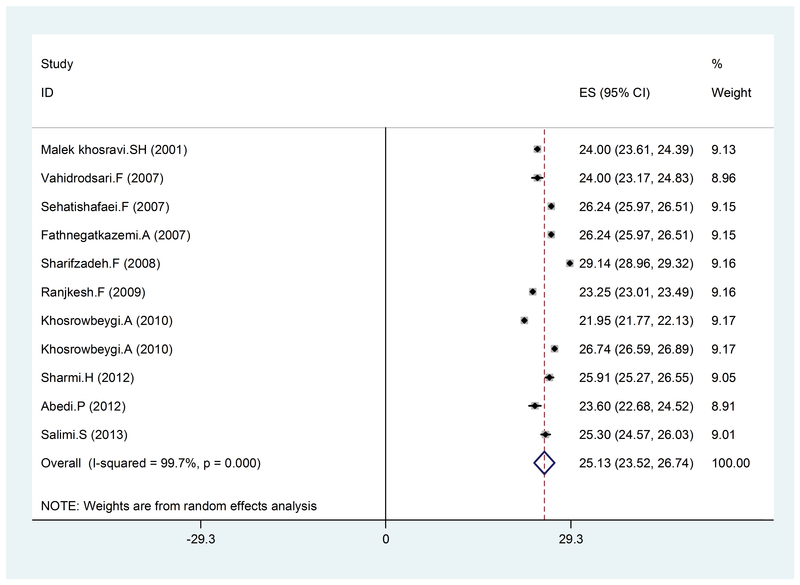
BMI in healthy pregnant women, by year, prevalence rate, and 95% CI interval. Each line segment shows the CI length. The diamond sign indicates the result of combining all studies at 95% CI.

**Figure 3:**
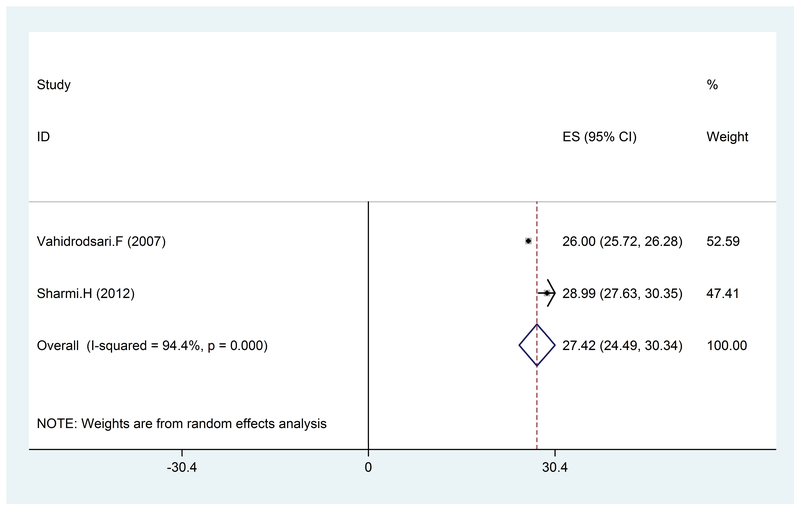
The BMI value in pregnant women with mild PE, by year, prevalence rate, and 95% CI. Each line segment shows the CI length. The diamond sign indicates the result of combining all studies at 95% CI.

**Figure 4:**
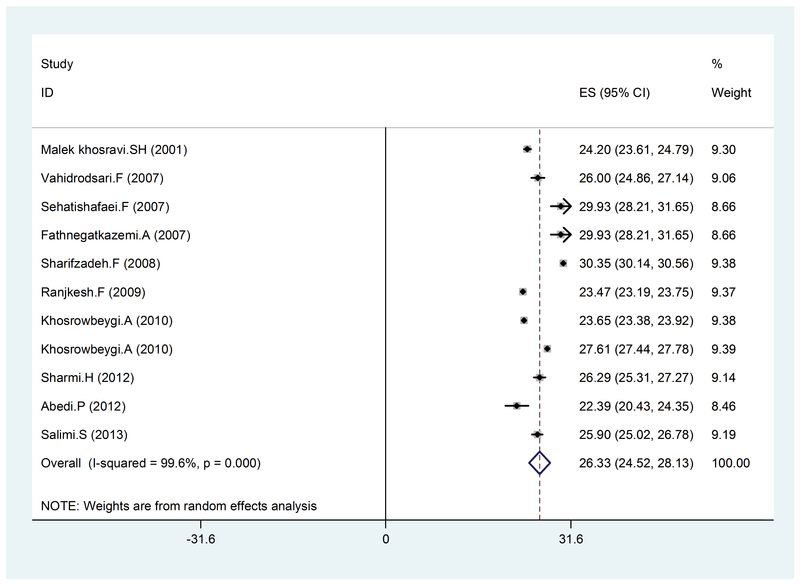
The BMI value in pregnant with severe PE, by year, prevalence rate, and 95% CI. Each line segment shows the CI length. The diamond sign indicates the result of combining all studies at 95% CI.

In the present study, a total of 16 articles which were published between 2000 and 2016 were reviewed and the total sample size was 5,946 women. The mean BMI was 25.13 (95% CI: 23.52-2.74) (Figure 2), 27.42 kg (95% CI: 24.4-30.34), (Figure 3), and 26.33 kg/m${}^{2}$ (95% CI: 24.52-28.13) (Figure 4) in the healthy, mild, and severe PE groups, respectively. As seen in Table II, the mean maternal age in the healthy and affected groups was 26.21 and 28.20 years, respectively, which indicates the effect of age on the disorder, considering the studied variable (BMI); however, this relationship was not significant. Also, the mean systolic and diastolic blood pressure was 142.75, 99.26 and 101.59, 70.76 mmHg in the affected and healthy groups, respectively, which indicates a direct relationship between hypertension and preeclampsia. These results revealed that hypertension is somewhat associated with an increase in BMI. Also, the average week of pregnancy in the healthy and affected mothers is 34.15 and 33.82 weeks, respectively, which seems to indicate no specific relationship between individuals with preeclampsia and BMI with the average week of pregnancy.

**Figure 5:**
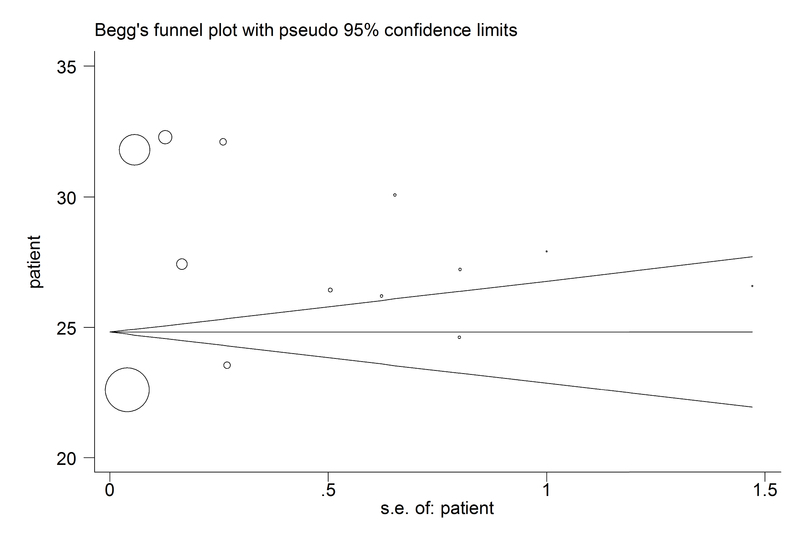
Beggs Funnel plot for publication bias.

According to the publication bias figure, Beggs test showed that the effect of bias in these studies was significant. (p = 0.584) (Figure 5).

**Table 2:** The mean of variables in the studies


**Variables**	**Number of study**	**95% confidence interval**
	Healthy group	13	26.21 (24.04-28.38)
Mean age of pregnant mothers	Affected group	2	28.20 (26.61-29.80)
		Healthy mothers	5	101.59 (96.60-106.58)
Mean systolic blood pressure	Affected mothers	5	142.75 (132.12-153.38)
		Healthy mothers	7	70.76 (65.87-75.65)
Mean diastolic blood pressure	Affected mothers	7	99.26 (91.74-106.79)
		Healthy mothers	8	34.15 (37.16-31.15)
Mean week of pregnancy	Affected mothers	8	33.82 (30.67-36.96)
		Healthy mothers	2	159.14 (157.04-161.24)
Mean maternal height (in cm)	Affected mothers	2	159.19 (157.90-160.47)
		Healthy mothers	2	3094.39 (2837.84-3350.92)
Mean maternal weight (gr)	Affected mothers	2	2508 (1903.35-3113.83)
		Healthy mothers	11	25.13 (23.52-26.74)
	Affected mothers (mild)	2	27.42 (24.49-30.34)
<brow>-3</erow> Mean BMI	Affected mothers (severe)	11	26.33 (24.52-28.13)
	Healthy mothers	8	34 (31-37)
Mean pregnancy week	Affected mothers	8	33 (30-37)
		Healthy mothers	5	101.50 (96.6-106.58)
Mean blood pressure systolic	Affected mothers	5	147.75 (132.12-153.38)
		Healthy mothers	7	56.24 (32.36-80.12)
Mean blood pressure diastolic 8	Affected mothers	7	81.49 (64.03-98.95)

## 4. Discussion

In this study, the average age of the mothers was 21.26 years in the healthy group and 20.28 years in the affected mothers. The average height of mothers was 159.14 cm in the healthy group and 159.19 cm in the affected group. Finally, the mean value of BMI was 25.13% in the healthy group and 26.33% in the (severely) affected group. The highest BMI in the healthy pregnant women was 29.14% and the lowest one was 21.95%. Furthermore, the highest BMI in the pregnant women with preeclampsia was 30.35% and the lowest was 22.39%. Moreover, in this study, the general prevalence of preeclampsia (severe) was 7% and the general prevalence of preeclampsia (mild) was obtained as 23%. The mean systolic hypertension in the studied group was 142.75 mmHg, while that of the healthy group was 101.59 mmHg, and the mean diastolic hypertension was 99.26 mmHg in the affected group and 70.76 mmHg in the healthy mothers.

According to the results obtained earlier, it can be concluded that the relationship between age and the incidence of intended disorder considering BMI was not significant. Further, there is no specific relationship between those with preeclampsia and BMI with the mean of gestational age.

Hypertension has a direct relationship with preeclampsia. According to these results, it can be concluded that hypertension is one of the factors that increases BMI in pregnant women. Additionally, high BMI has a relationship with preeclampsia in pregnant women. This result is relatively consistent with Brown's study; in the Brown's study, the percentage of preeclampsia prevalence (4.5%) in women with light weight is approximately half of the women with normal weight (0.09) (31).

In a study by Vahid Roodsari *et al.* BMI was reported to be 24 kg/m${}^{2}$ in the pre-pregnancy control group, 26.124 kg/m${}^{2}$ in the gestational hypertension group, 26.24 kg/m${}^{2}$ in the group with mild preeclampsia and 26.24 kg/m${}^{2}$ in the severe preeclampsia group. It has been reported (32), according to the obtained results, that if weight increases before pregnancy, weight loss will prevent mortality during pregnancy. Jang *et al.* in Seoul, Korea, compared two groups and showed that the incidence of preeclampsia in women with overweight women is higher than those of normal weight (33). Derif *et al*. also stated that hypertension problems occurs more in overweight women indicating its incidence 7% to 17% (34). Kramer, in a researcher at the University of New York, found that as the BMI increases among the mothers, the risk of cesarean operation will increase significantly (35).

In the study by Ohkuchi *et al.* the incidence of hypertension during pregnancy in women with less weight, normal weight, weight more than normal, and obese was 1.1%, 1.8%, 5.8%, and 19.6%, respectively, which besides its consistency with the results of this study, indicates that the incidence of pre-pregnancy hypertension in obese women significantly increases (36). Sibai also reported in a study that high weight before pregnancy is an important risk factor for pregnancy toxicity (37). These results are relatively consistent with the conducted study. In addition, in a study by Bondar in the United States, it was shown that 56% of women with gestational toxicity were suffering from either overweight or obesity, and every 4-unit increase in BMI was related to twice the risk of gestational toxicity and an 8-unit increase in BMI was related to three times the risk of gestational toxicity (38). In another study by Belogolovkin *et al.* in the United States, it was concluded that the probability of a pregnancy-related hypertention in women with a high BMI (26.1-29 kg/m${}^{2}$) was 6%, whereas in women with a normal BMI (19.86-26 kg/m${}^{2}$) it was 3.2%, and the probability of pregnancy toxicity in women with normal BMI is 1.9%, whilein women with a high BMI, it was 2.8% (39).

Cogswell and Yip, in the United States, showed that women with high BMI have more deliveries (40). In the conducted study also women with heavy weight had more deliveries.

Naevy *et al.*, found that the prenatal mortality was higher in women with heavy weight than other groups, and it is said that the reason behind it lies in the increased multiple pregnancy and preterm birth (41).

Women with high BMI have adverse effects of pregnancy, delivery, and neonate, and even in the pre-pregnancy period; women with abnormal weight face with problems such as polycystic ovary, infertility, and failure in infertility treatments (41). Pregnant women with abnormal weight and obesity are subject to the risk of diabetes mellitus type 2, gestational diabetes, and preeclampsia (42), thromboembolic diseases (43), dyspnea (44), asthma (45), cholecystitis (46), back pain, pelvic pain (47) and urinary incontinence (48).

### Problems regarding data extraction

Among the problems we encountered during the data extraction in this article were the irrelevancy of the article to the stated subject, the inaccessibility of the full text of the article, and the incomplete information given in the abstract part of the article. The control of all discomfit factors was not possible, and factors such as diet are effective factors that have not been measured in studies.

## 5. Conclusion

This meta-analysis study, in which BMI was evaluated, showed that the risk of preeclampsia may increase with an increase in BMI. Therefore, BMI can be considered as one of the ways to diagnose the preeclampsia.

##  Conflict of Interest

The authors declare that there is no conflict of interest.
